# The potential of *Rhodobacter sphaeroides* extract as an alternative supplement for cell culture systems

**DOI:** 10.1128/spectrum.02456-23

**Published:** 2024-02-06

**Authors:** Subin Lee, Yang-Hoon Kim, Jiho Min

**Affiliations:** 1 Department of Bioprocess Engineering, Jeonbuk National University, Jeonju, Jeonbuk, South Korea; 2 School of Biological Sciences, Chungbuk National University, Cheongju, South Korea; Suranaree University of Technology, Meung, Thailand

**Keywords:** cell culture supplement, *Rhodobacter sphaeroides*, alternative to fetal bovine serum, cell growth and viability, oxidative stress mitigation

## Abstract

It is essential to identify suitable supplements that enhance cell growth, viability, and functional development in cell culture systems. The use of fetal bovine serum (FBS) has been common, but it has limitations, such as batch-to-batch variability, ethical concerns, and risks of environmental contamination. In this study, we explore the potential of *Rhodobacter sphaeroides* extract, derived from a probiotic photosynthetic bacterium, as an alternative supplement. Our results demonstrate that the extract from *R. sphaeroides* significantly improves various aspects of cell behavior compared to serum-free conditions. It enhances cell growth and viability to a greater extent than FBS supplementation. Additionally, the extract alleviates oxidative stress by reducing intracellular levels of reactive oxygen species and stimulates lysosomal activity, contributing to cellular processes. The presence of abundant amino acids, glycine and arginine, in the extract may play a role in promoting cell growth. These findings emphasize the potential of *R. sphaeroides* extract as a valuable supplement for cell culture, offering advantages over the use of FBS.

**IMPORTANCE:**

The choice of supplements for cell culture is crucial in biomedical research, but the widely used fetal bovine serum (FBS) has limitations in terms of variability, ethics, and environmental risks. This study explores the potential of an extract from *Rhodobacter sphaeroides*, a probiotic bacterium, as an alternative supplement. The findings reveal that the *R. sphaeroides* extract surpasses FBS in enhancing cell growth, viability, and functionality. It also mitigates oxidative stress and stimulates lysosomal activity, critical for cellular health. The extract’s abundance of glycine and arginine, amino acids with known growth-promoting effects, further highlights its potential. By providing a viable substitute for FBS, the *R. sphaeroides* extract addresses the need for consistent, ethical, and environmentally friendly cell culture supplements. This research paves the way for sustainable and reliable cell culture systems, revolutionizing biomedical research and applications in drug development and regenerative medicine.

## INTRODUCTION

*Rhodobacter sphaeroides*, a purple non-sulfur bacterium, is known to produce a wide array of bioactive compounds, including antioxidants, antimicrobial agents, and growth-promoting factors (1). *R. sphaeroides* demonstrates enhanced antioxidant activity by increasing the activity of antioxidant enzymes, such as superoxide dismutase, catalase, and glutathione peroxidase (2). Additionally, it has the capability to produce high-value bioactive compounds, including coenzyme Q10, carotenoids, bacteriochlorophyll, and other valuable antioxidants. These bioactive compounds have demonstrated promising effects in various biological systems, such as improving wound healing, enhancing immune responses, and protecting against oxidative stress (1). Cell culture serves as a crucial tool in biomedical research, providing a platform for studying cellular behavior, disease mechanisms, and drug discovery. Optimal culture conditions and the provision of appropriate nutrients and growth factors are essential for successful cell culture experiments, ensuring cell growth, viability, and functional development.

Traditionally, fetal bovine serum (FBS) has been extensively utilized as a supplement in cell culture media. FBS comprises a complex mixture of proteins, hormones, growth factors, and other bioactive molecules that facilitate cell proliferation and survival (3). However, the use of FBS is associated with various drawbacks, including batch-to-batch variability, ethical concerns, and the potential risk of introducing contaminants into the culture system (4, 5). Additionally, FBS is derived from bovine fetuses, and its composition may vary depending on the manufacturing process and source, posing challenges in maintaining consistency across experimental results. Moreover, its relatively high cost can be a significant financial burden, particularly in large-scale cell culture applications (6). These limitations have fostered the quest for alternative supplements capable of providing consistent and reliable support for cell growth and function.

We hypothesized that supplementation with *R. sphaeroides* extract would enhance cell growth and viability by providing specific nutrients and growth factors essential for cell proliferation. Moreover, we expected the extract’s antioxidant properties to reduce intracellular oxidative stress, creating a favorable cellular environment for growth and development (7, 8). Additionally, considering the critical role of lysosomes in cellular processes, we hypothesized that *R. sphaeroides* extract could influence lysosomal activity, contributing to improved cellular functions and maintenance (9, 10). Understanding the potential benefits of *R. sphaeroides* extract supplementation in cell culture systems holds significant importance. By addressing the limitations associated with FBS, *R. sphaeroides* extract offers an alternative approach to enhance experimental consistency, mitigate ethical concerns, and improve the reliability of cell-based studies (5). Furthermore, unraveling the underlying mechanisms and identifying specific bioactive compounds within the *R. sphaeroides* extract will provide valuable insights into its mode of action and potential applications across diverse cell culture systems.

To investigate these hypotheses, we conducted a series of experiments comparing the effects of *R. sphaeroides* extract supplementation with those of FBS and serum-free conditions. We evaluated cell growth rates, viability, intracellular reactive oxygen species (ROS) levels, and lysosomal activity in HS27 cells treated with varying concentrations of *R. sphaeroides* extract. This study aimed to explore the potential benefits of *R. sphaeroides* extract as a supplement for cell culture systems, with a specific focus on its effects on HS27 cells. The primary objective was to assess the influence of *R. sphaeroides* extract on cell growth, viability, intracellular oxidative stress, and lysosomal activity. By examining these parameters, we aimed to determine the advantages of *R. sphaeroides* extract as a valuable supplement for optimizing cell culture conditions and improving the reliability and reproducibility of cellular experiments in various biomedical research fields.

## RESULTS

### Growth-stimulating potential of Rbs^Aero^ and Rbs^Anaero^ on HS27 cell growth

**Fig 1:**
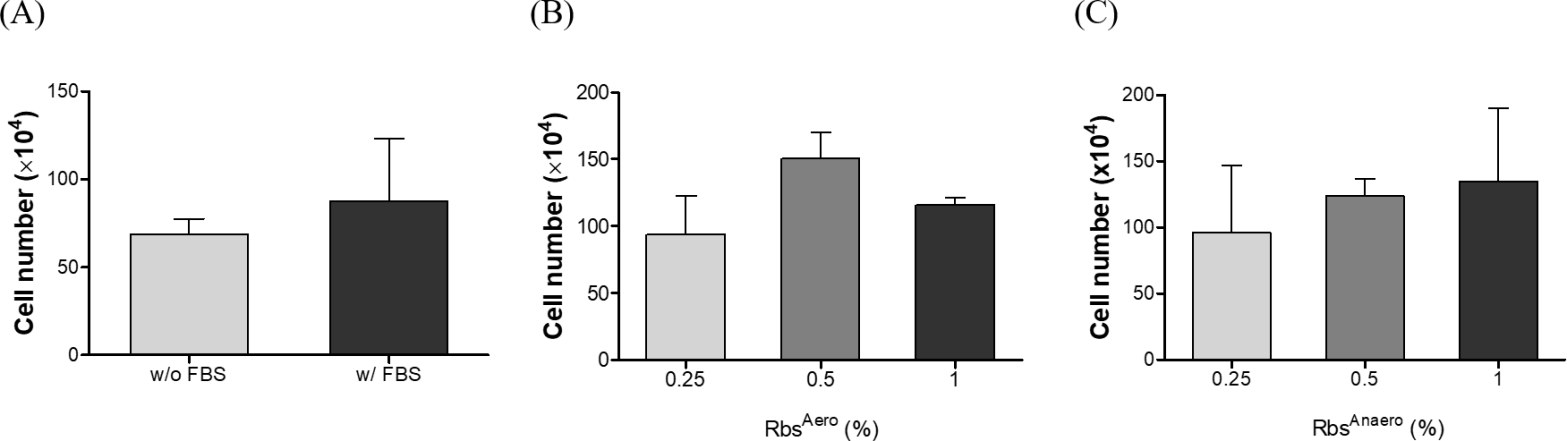
HS27 cell number based on the FBS and *R. sphaeroides* extract treatment. FBS was treated at 0.5%, and *R. sphaeroides* was treated at 0.25%, 0.5%, and 1%. The error bar represented 95% confidence intervals. (A) Comparison of cell numbers with and without FBS added. (B) Cell number by the concentration of Rbs^Anaero^. (C) Cell number by the concentration of Rbs^Aero^.

Before confirming the serum replacement effect of extraction after *R. sphaeroides* culture, the effect of FBS, a serum commonly used for mammalian cell culture, on cell growth was investigated. In this study, human fibroblasts (HS27 cells) were cultured in a medium with and without 0.5% FBS, as well as in a serum-free medium, and then cell numbers were measured and compared. The result confirmed that the addition of 0.5% FBS increased the cell number by approximately 1.3 times compared to the condition without the addition of serum (Fig. 1).

The extract of *R. sphaeroides* appeared red when cultured under aerobic dark conditions and yellow when cultured under anaerobic light conditions. These extracts were named Rbs^Aero^ and Rbs^Anaero^, respectively. They were added at concentrations of 0.25%, 0.5%, and 1% as substitutes for FBS in the culture medium of HS27 cells to evaluate their effects on cell growth. The results showed that the addition of Rbs^Aero^, the extract from *R. sphaeroides* cultured under aerobic dark conditions, significantly increased the number of HS27 cells compared to the addition of 0.5% FBS at all concentrations tested. Among the concentrations used, 0.5% Rbs^Aero^ stimulated cell growth the most (Fig. 1B). Additionally, compared to cells grown in a serum-free medium, the number of HS27 cells increased approximately twofold when 0.5% Rbs^Aero^ was added. Furthermore, it increased by 1.7 times compared to the addition of 0.5% FBS, indicating that Rbs^Aero^ at this concentration can effectively replace FBS and promote cell growth. Moreover, in the case of Rbs^Anaero^, the number of cells increased in a concentration-dependent manner as the concentration of Rbs^Anaero^ increased (Fig. 1C). Thus, it was confirmed that the extract isolated from *R. sphaeroides* cultured under two different conditions exhibited the ability to stimulate HS27 cell growth, suggesting its potential as a substitute for the previously used FBS.

### Inhibition of intracellular ROS generation by *R. sphaeroides* extracts and their antioxidant activity

**Fig 2:**
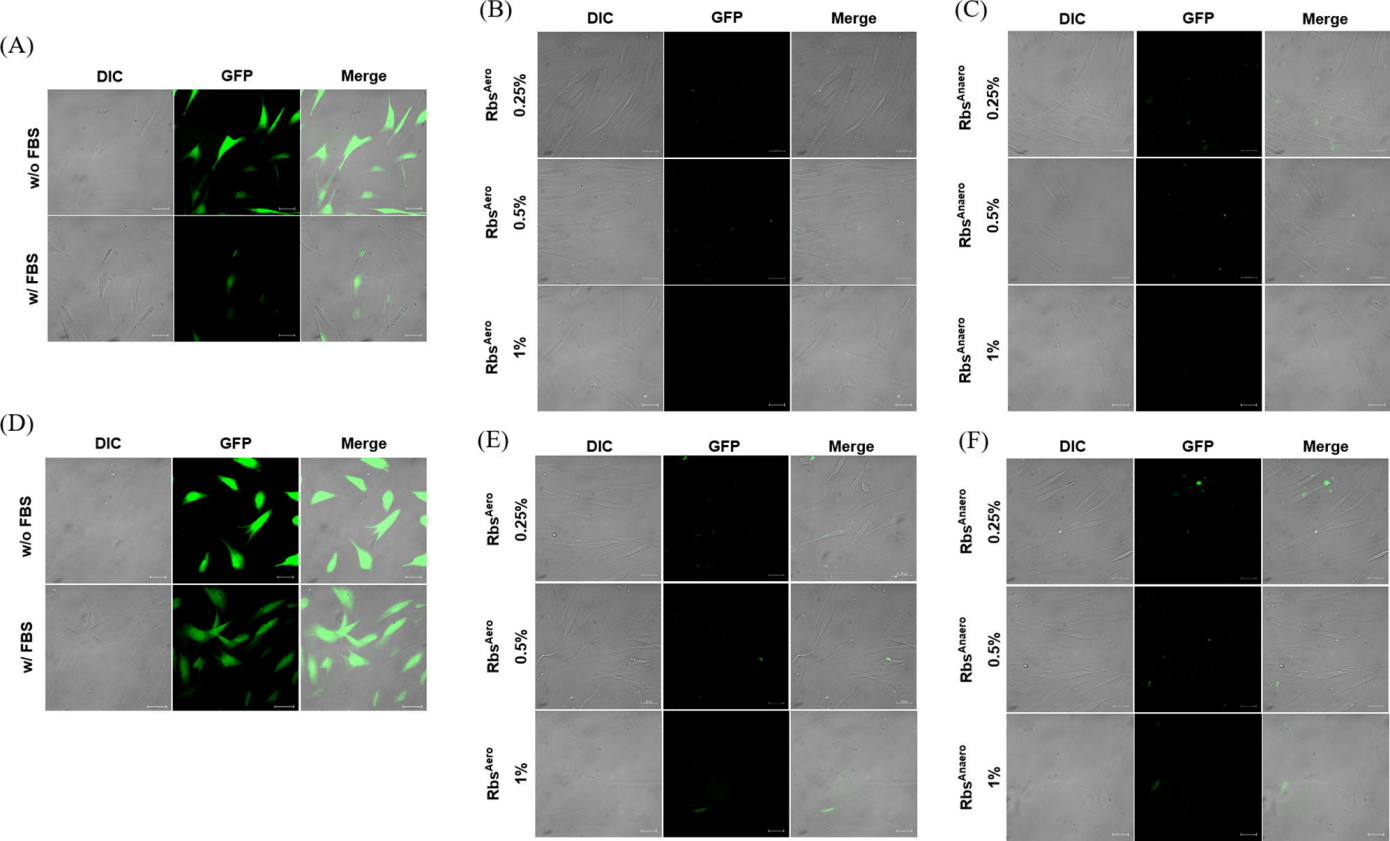
Comparison of ROS fluorescence generated in HS27 cells and ROS inhibition by H_2_O_2_ through *R. sphaeroides* extract treatment. Merged image is a combination of differential interference contrast (DIC) and green fluorescent protein (GFP) images. The scale bar represents 20 µm. (A–C) Cellular ROS fluorescence induced by FBS and Rbs extracts. (D–F) Cellular ROS fluorescence induced by H_2_O_2_ in the presence of FBS and Rbs extracts.

The effects of Rbs^Aero^ and Rbs^Anaero^ on the intracellular environment of HS27 cells were compared and analyzed by fluorescent staining to assess the generation of ROS, which can occur in unfavorable growth conditions (Fig. 2A through C). The control group without FBS or *R. sphaeroides* extracts exhibited the largest amount of ROS generation, while the 0.5% FBS treatment condition showed a significant but lower level of ROS compared to the control group. In contrast, HS27 cells treated with Rbs^Aero^ and Rbs^Anaero^ showed no observable ROS generation. ROS components generated in cells induce oxidative stress, negatively affecting growth. Although FBS slightly reduced ROS generation, Rbs^Aero^ and Rbs^Anaero^ did not exhibit ROS inhibitory activity.

To investigate the antioxidant activity of the *R. sphaeroides* extract, HS27 cells were pretreated with 40 µM H_2_O_2_ to artificially induce higher levels of ROS (Fig. 2D through F). Similar to Fig. 2A, untreated HS27 cells showed a substantial amount of ROS. However, treatment with 0.5% FBS resulted in a slight decrease in fluorescence compared to the untreated cells. Remarkably, the HS27 cells treated with Rbs^Aero^ and Rbs^Anaero^ exhibited minimal ROS fluorescence even after the induction of a high amount of ROS by pretreatment with 40 µM H_2_O_2_. This confirmed the antioxidant activity of the *R. sphaeroides* extract in reducing both internally generated ROS and externally introduced ROS-inducing substances.

**Fig 3:**
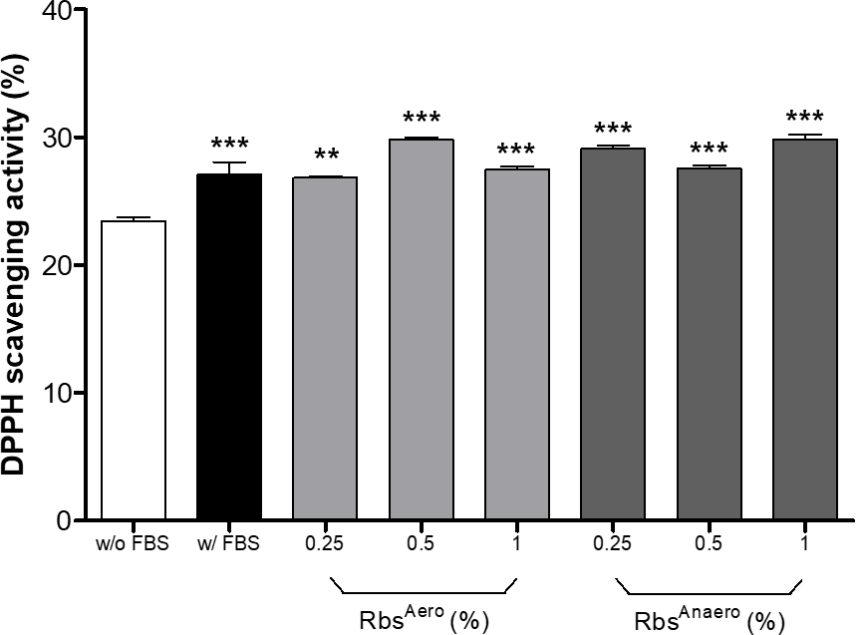
Comparison of intracellular antioxidant activity of *R. sphaeroides* extract treatment concentration. After *R. sphaeroides* treatment, extraction from HS27 cells was performed to measure antioxidant activity by using DPPH scavenging activity. Significantly different without the FBS group (**P < 0.01, ***P < 0.001, one-way ANOVA).

Furthermore, the antioxidant activity of HS27 cells cultured with *R. sphaeroides* extract instead of FBS was examined using the 2,2-diphenyl-1-picrylhydrazyl (DPPH) scavenging activity assay (Fig. 3). Cells cultured with FBS displayed higher antioxidant activity compared to cells cultured without FBS. When comparing the antioxidant activity of Rbs^Aero^-treated cells, similar or higher activity was observed at a treatment concentration of 0.5%. Moreover, Rbs^Anaero^-treated cells exhibited higher antioxidant activity at all treatment concentrations compared to the control group. These findings highlight the potential of *R. sphaeroides* extract to enhance the intracellular antioxidant activity in HS27 cells.

### Enhanced intracellular lysosomal activity in HS27 cells through *R. sphaeroides* extract treatment

**Fig 4:**
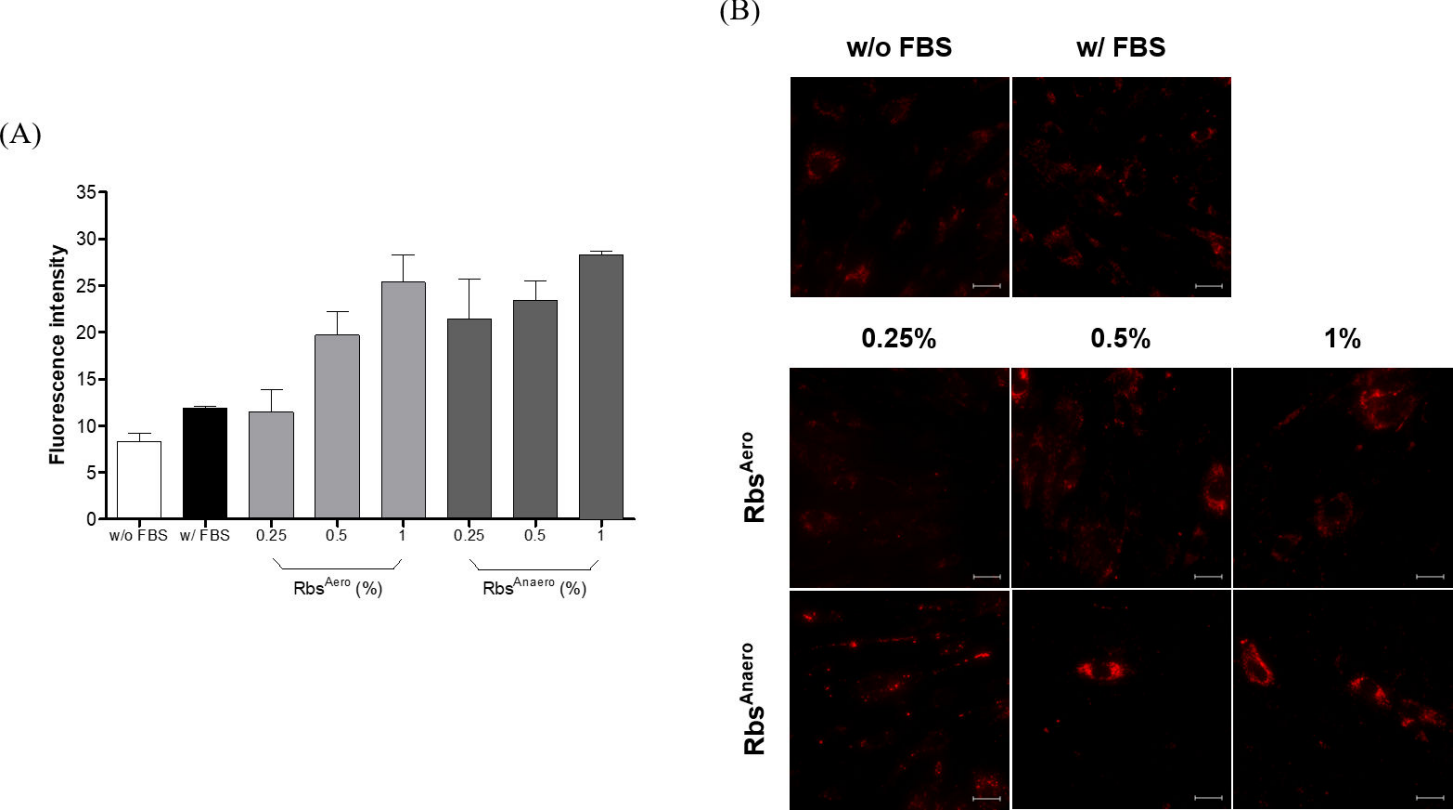
Measurement of lysosomal fluorescence by treatment concentration of *R. sphaeroides* extract. Comparison of activity through fluorescence after lysosomal staining with 100 nM LysoTracker Red DND-99. (**A**) The partial lysosomal fluorescence image. The scale bar represents 20 µm. (**B**) Total intracellular lysosomal fluorescence intensity. The error bar represents 95% confidence intervals.

Intracellular lysosomes play a central role in immune responses, being responsible for processing invading bacteria, viruses, and aged organelles. This study aimed to analyze the changes in intracellular lysosomal activity when treating HS27 cells with *R. sphaeroides* extract as a substitute for FBS. Similar to the previous experiment, HS27 cells were treated with 0.25%, 0.5%, and 1% concentrations of *R. sphaeroides* extracts obtained from aerobic dark and anaerobic light cultures. Lysosomal fluorescence was visualized through lysotracker staining (Fig. 4).

Lysosomal fluorescence images showed no significant difference between cells treated with and without FBS. However, when cultured with FBS, a slight lysosomal fluorescence signal was observed within HS27 cells. In contrast, in the case of HS27 cells cultured with Rbs^Aero^ and Rbs^Anaero^ instead of FBS, red fluorescence signals appear as shown in the figure. Furthermore, it was observed that the lysosomal fluorescence intensity increased with increasing treatment concentrations under both Rbs^Aero^ and Rbs^Anaero^ conditions. Notably, Rbs^Anaero^ exhibited stronger fluorescence compared to Rbs^Aero^. This indicates that treatment with *R. sphaeroides* extract can enhance lysosomal activity in cells, with slightly higher fluorescence observed in Rbs^Anaero^-treated cells compared to Rbs^Aero^-treated cells.

Additionally, the lysosomal fluorescence intensity of whole cells was quantitatively measured using HS cells and then compared. As shown in Fig. 4B, similar to Fig. 4A, increased fluorescence intensity was observed in HS cells cultured with FBS treatment compared to the untreated control group. Comparing cells treated with *R. sphaeroides* extract to untreated cells, stronger fluorescence intensity was detected in all conditions except for HS cells treated with 0.25% Rbs^Aero^. The highest fluorescence intensity was measured in Rbs^Anaero^ at a concentration of 1%, suggesting greater lysosomal activity under anaerobic conditions compared to aerobic conditions. Additionally, both conditions exhibited concentration-dependent lysosomal activity, indicating that the intracellular lysosomal activity was increased with *R. sphaeroides* extract treatment.

### Amino acid analysis by *R. sphaeroides* extracts implicating HS27 cell growth stimulation

To investigate the potential growth-stimulating effects of *R. sphaeroides* extracts on HS27 cells, in addition to their antioxidant activity, the amino acid composition of the extracts was analyzed. The analysis included *R. sphaeroides* extracts obtained from aerobic culture under dark conditions (Rbs^Aero^) and anaerobic culture under light conditions (Rbs^Anaero^) (Table 1). In both Rbs^Aero^ and Rbs^Anaero^ conditions, glycine was found to be the most abundant amino acid. In Rbs^Aero^, glutamic acid, alanine, aspartic acid, and arginine were produced in descending order. Conversely, in Rbs^Anaero^, the overall amino acid production was lower compared to Rbs^Aero^, but the production levels of arginine, glutamic acid, and histidine were relatively high after glycine. Based on the amino acid analysis results, it was observed that glycine and arginine were potentially beneficial for cell growth when treating HS27 cells with *R. sphaeroides* extracts obtained under the two culture conditions.

**TABLE 1:** Measurement results of free amino acids in the *R. sphaeroides* extract^*a*^

Amino acid	Rbs^Aero^	Rbs^Anaero^
Glycine	51.0	7.0
Alanine	25.0	1.0
Serine	0.0	1.0
Proline	0.0	0.0
Valine	0.0	0.0
Threonine	3.0	1.0
Leucine	0.0	0.0
Isoleucine	0.0	0.0
Asparagine	1.0	1.0
Aspartic acid	8.0	4.0
Lysine	0.0	0.0
Glutamine	0.0	0.0
Glutamic acid	39.0	5.0
Methionine	1.0	0.0
Histidine	5.0	5.0
Phenylalanine	0.0	0.0
Arginine	6.0	6.0
Tyrosine	2.0	1.0
Tryptophan	0.0	0.0
Cysteine	3.0	3.0

^*a*^ Rbs^Aero^ represents the amino acid content under aerobic dark conditions, while Rbs^Anaero^ represents the amino acid content under anaerobic light conditions. Concentration units are expressed in ppb.

## DISCUSSION

In this study, we investigated the effect of an extract derived from *R. sphaeroides*, a probiotic photosynthetic bacterium, on the growth and intracellular antioxidant activity of HS27 cells, a type of animal cell. We aimed to evaluate the potential of *R. sphaeroides* extract as a substitute for serum, which is a crucial and expensive component in animal cell culture. Our results confirmed that the addition of FBS, a type of serum, to the cell culture medium significantly increased cell growth compared to serum-free conditions, consistent with previous studies highlighting the essential role of serum components, such as growth factors and nutrients, in promoting cell proliferation (3). Interestingly, when the *R. sphaeroides* extract was added to the medium, we observed even higher cell growth rates compared to FBS supplementation at the same concentration. The *R. sphaeroides* extract used in this study was isolated through aerobic culture under dark conditions and anaerobic culture under light conditions. The number of HS27 cells exhibited a concentration-dependent increase when treated with Rbs^Anaero^ (Fig. 1B), while Rbs^Aero^ showed the highest cell growth at a concentration of 0.5% (Fig. 1C). These findings suggest that the *R. sphaeroides* extract potentially contains bioactive compounds that enhance cell proliferation in HS27 cells by providing specific nutrients or growth factors that are beneficial for cell growth. Furthermore, Rbs^Aero^ and Rbs^Anaero^ generally showed higher cell viability compared to the FBS group, indicating that the *R. sphaeroides* extract not only promotes cell growth but also improves cell viability and overall cell health. These results underscore the potential of *R. sphaeroides* extract as a valuable supplement for cell culture systems.

ROS plays an important role in cellular processes, and excessive ROS accumulation can cause oxidative stress and negatively affect cell growth (7, 8, 11). In our study, we evaluated the effect of *R. sphaeroides* extract on intracellular ROS levels in HS27 cells. We found that treatment with *R. sphaeroides* extract resulted in reduced ROS fluorescence compared to the untreated control, indicating its ability to mitigate oxidative stress during cell growth (Fig. 2A, B, and C). Additionally, when HS27 cells were exposed to excessive ROS stress induced by H_2_O_2_, the addition of *R. sphaeroides* extract effectively attenuated ROS fluorescence and exhibited antioxidant activity, improving the cellular environment for growth (Fig. 2D, E, and F). These results suggest that *R. sphaeroides* extract possesses antioxidant properties that can positively impact cell growth and viability by reducing intracellular oxidative stress (12). To support these findings, we also analyzed the intracellular antioxidant activity of HS27 cells, which showed that both the FBS and *R. sphaeroides* extract treatment increased antioxidant activity compared to the control group (Fig. 3). Particularly, Rbs^Anaero^ exhibited higher antioxidant activity at all treatment concentrations than FBS and Rbs^Aero^, indicating that the anaerobically grown *R. sphaeroides* extract contained more antioxidant compounds than Rbs^Aero^ (13, 14). However, it was observed that both culture conditions provided a significant increase in antioxidant activity in HS27 cells. Processing with Rbs extract prevented the excessive generation of oxidative substances, thereby aiding in the preservation of cell structure and function (15, 16). The increased intracellular antioxidant effect may contribute to the prevention of DNA damage from oxidative stress and enhance protection for immune system cells, reducing intracellular inflammation and the generation of free radicals (17). These findings suggest that Rbs extract treatment can reduce intracellular oxidative stress, inhibit the excessive generation of oxidants, and enhance antioxidant defense mechanisms.

Lysosomes, as important organelles involved in various cellular processes including degradation and recycling, play a crucial role in cellular homeostasis (9, 18, 19). In our study, we investigated the effect of *R. sphaeroides* extract on lysosomal activity in HS27 cells. Our results demonstrated that treatment with *R. sphaeroides* extract increased lysosomal fluorescence intensity compared to the control group (Fig. 4). The enhanced lysosomal activity suggests that *R. sphaeroides* extract treatment can promote cellular processes related to lysosomal function, potentially benefiting cell growth and maintenance (18, 20). Furthermore, when analyzing the lysosomal fluorescence intensity of whole cells, we observed stronger fluorescence in the presence of FBS compared to the serum-free condition. However, treatment with *R. sphaeroides* extract showed a significantly higher fluorescence intensity compared to the FBS treatment group, indicating its ability to enhance overall lysosomal activity in HS27 cells. Specifically, Rbs^Anaero^ exhibited the highest lysosomal activity, suggesting that *R. sphaeroides* extract can stimulate the growth of animal cells by enhancing lysosomal activity, which is crucial for maintaining cellular homeostasis. During the treatment with *R. sphaeroides* extract, lysosomes are activated to facilitate the breakdown of unnecessary cellular components into nutritents such as amino acids, sugars, and lipids. By integrating and transmitting this nutritional information, lysosomes contribute to the homeostasis of cells and organisms. Furthermore, through the process of autophagy, lysosomes play a crucial role in breaking down cellular constituents, contributing to the maintenance of cell health and supporting growth (21, 22).

In order to identify the specific components in *R. sphaeroides* extract that contribute to the stimulation of HS27 cell growth, we conducted an amino acid analysis of the extract. The analysis revealed that glycine was the most abundant amino acid in both aerobic and anaerobic conditions, followed by arginine, which was the second most abundant in Rbs^Anaero^ (Table 1). These amino acids, glycine and arginine, are known to play important roles in cell metabolism, protein synthesis, and cell signaling, and may contribute to the observed growth-promoting effect of *R. sphaeroides* extract. Glycine, an essential amino acid for protein synthesis, supports fibroblast growth and tissue regeneration (23). It also possesses anti-inflammatory properties, inhibits cytotoxic substance production, and promotes fibroblast growth (24). Moreover, glycine is a crucial component of collagen, the primary fibrous protein synthesized by fibroblasts, and its adequate supply is essential for collagen fibrillation, tissue regeneration, and promotion of fibroblast growth (25–27). Arginine, on the other hand, is essential for protein synthesis and supports the growth and regeneration of fibroblasts. It facilitates blood circulation, aiding in the delivery of nutrients and oxygen (28, 29). Arginine also helps regulate inflammation and promotes fibroblast growth by reducing the inflammatory response (30). Additionally, it plays a role in collagen synthesis, contributing to tissue strength and elasticity (27). These findings suggest that the *R. sphaeroides* extract may affect the growth of animal cells by providing an abundant supply of glycine and arginine, which have known growth-promoting effects. Glutamic acid is considered an essential amino acid crucial for the survival and growth of cells in cell culture, including fibroblasts and human cells. It is synthesized from glucose and plays a vital role in protein synthesis, contributing to the growth and maintenance of cells (31–33). Cysteine serves a protective role against oxidative stress and possesses strong antioxidant effects, contributing to cell stability and facilitating growth and function in suitable environments. Consequently, it enhances cellular resilience. Insufficient cysteine levels may negatively impact molecules such as glutathione and taurine, which are essential for antioxidant defense. This deficiency can induce harmful oxidative stress, diminishing the survival capability of cells (34, 35).

The potential effects on other animal cell models, not limited to HS27 cells, suggest applicability and potential benefits across various animal cell types. Lee et al. (36) presented the effects and characteristics of *R. sphaeroides* extract on the growth of HaCaT cells. Additionally, An et al. (37) evaluated the protective capacity of *R. sphaeroides*, attributed to its antioxidant effects, on a Caco-2 cell line model.

In conclusion, our study demonstrates that the addition of *R. sphaeroides* extract to HS27 cell cultures improves cell growth, viability, and lysosomal activity compared to FBS supplementation. Although further investigation is required to identify the specific bioactive compounds and underlying mechanisms responsible for the observed effects of *R. sphaeroides* extract on cell growth and intracellular development, the extract exhibits antioxidant properties and has the potential to reduce oxidative stress induced by ROS and H_2_O_2_. Furthermore, the presence of abundant glycine and arginine in the *R. sphaeroides* extract suggests its potential as a serum substitute for promoting animal cell growth. Based on these experimental results, *R. sphaeroides* extract, a probiotic photosynthetic bacterium that can be easily cultivated, emerges as a promising alternative to conventional FBS supplementation, highlighting its value as a valuable supplement for cell culture systems.

## MATERIALS AND METHODS

### Preparation of *R. sphaeroides* extract

For intracellular material extract experiments, *R. sphaeroides* KCTC 1434 (Korean Collection for Type Cultures) was cultivated in SIST medium (Sistrom’s Minimal Medium A) under aerobic conditions without light and anaerobic conditions with light (38, 39). Under anaerobic conditions, the culture was incubated after nitrogen purging for 15 minutes. Following a 48-hour seed culture, the main culture was initiated and grown at 30°C and 180 rpm until it reached the stationary phase. Upon cell growth, pellets were obtained by centrifugation, dissolved in distilled water, and subjected to sonication for 25 minutes (on:off = 30 seconds:59 seconds) using a 20% pulse (13). Subsequently, cell debris was removed by centrifugation at 13,000 rpm for 10 minutes, and intracellular substances were extracted using a 0.2-µm syringe filter (36). This process allowed for the experimental extraction of intracellular substances. Cultures grown under aerobic dark conditions were denoted as Rbs^Aero^, while those grown under anaerobic light conditions were denoted as Rbs^Anaero^. *R. sphaeroides* cultured under aerobic dark conditions was diluted based on the cell number in the stationary phase under anaerobic light conditions, which exhibited relatively slower growth. When the 200-mL culture medium reached the stationary phase, the extracted *R. sphaeroides* was defined as 100%.

### Culture and count of HS27 cell

HS27 (ATCC CRL-1634) cells, derived from human fibroblasts, were cultured in Dulbecco’s Modified Eagle’s Medium (DMEM) supplemented with 0.5% FBS and penicillin-streptomycin. Cell numbers were determined by culturing for 24 hours in 100-nm culture dishes containing DMEM with or without 0.5% FBS. Subsequently, *R. sphaeroides* extract was added to the medium at concentrations of 0.25%, 0.5%, and 1% as a substitute for FBS, and the cells were further cultured. To quantify the HS27 cell number, the cells were washed twice with phosphate-buffered saline and were detached by treating them with 1× trypsin EDTA for 1 minute and 30 seconds. After centrifugation, the cells were stained with trypan blue at a 1:1 ratio, and the number of viable cells was determined using a hemocytometer.

### Measurement of ROS fluorescence

To measure the ROS fluorescence generated in HS27 cells in response to *R. sphaeroides* treatment, HS27 cells were cultured in a six-well plate at a concentration of 1 × 10^5^ cells/well on a cover glass for 24 hours. Subsequently, the cells were treated with 400 µM H_2_O_2_ and two types of *R. sphaeroides* extract at various concentrations for 24 hours. Following the treatment, 10 µM of 2’,7’-dichlorodihydrofluorescein diacetate (DCFH-DA) fluorescent reagent was added to the cells and incubated at 37°C without light for 2 hours. After washing the cells with Dulbecco's phosphate-buffered saline (DPBS), the fluorescence was measured and analyzed using a fluorescence microscope.

### Lysosomal fluorescence activity

To measure the lysosomal activity in HS27 cells in response to varying concentrations of *R. sphaeroides* extract, HS27 cells were cultured on a cover glass at a concentration of 1 × 10^5^ cells/well for 24 hours. Following 24 hours of treatment with each concentration of *R. sphaeroides* extract, 100 nM of LysoTracker Red DND-99 fluorescent reagent was added and incubated for 20 minutes in the absence of light. After washing with DPBS, the cells were imaged using a fluorescence microscope, and the images were processed using the Zeiss image browser software. Furthermore, to confirm the enhancement of antioxidant activity in lysosomes after treatment with *R. sphaeroides* extract, HS27 cells were cultured in a 150-mm culture dish. The cells were collected by dispensing lysis buffer and scraping the cells using a cell scraper. The obtained cell suspension was vortexed for 5 minutes at 10-second intervals, and the supernatant was collected after centrifugation at 500 × *g* for 5 minutes. Subsequently, the obtained supernatant was further centrifuged at 20,000 × *g* for 30 minutes to collect the lysosomes.

### Antioxidant activity

To assess the antioxidant activity of lysosomes, the DPPH scavenging assay was performed. The experimental procedure followed Dojindo’s DPPH assay protocol, in which the DPPH solution was prepared by dissolving it in ethanol to a concentration of 0.2 mM. The sample and the DPPH reagent were mixed in a 1:1 ratio and allowed to react for 30 minutes at room temperature in the absence of light. Subsequently, the absorbance was measured at 517 nm using a microplate reader.

### Amino acid analysis

*R. sphaeroides* extract was measured by liquid tandem mass chromatography (LC/MS/MS). A UPLC system (Acquity System, Waters, Milford, USA) was coupled to a Xevo TQ-S triple quadrupole mass spectrometer (Waters). The UPLC column was lmtakt Intrada Amino Acid C18 (50 × 2 mm, 3 µm). The mobile phase buffer A was ACN (acetonitrile):100 mM ammonium formate = 20:80 (vol/vol), and buffer B was ACN:THF:25 mM ammonium formate:formic acid = 9:75:16:0.3 (vol/vol/vol/vol). The flow was 0.4 mL/min and established a gradient from 0 to 100% for the buffer B for 17 minutes.

### Data analysis

All experiments were performed using three independent samples measured simultaneously to ensure robustness and enable error analysis. The resulting averages were calculated, and the corresponding correlations and standard deviations were determined for the various experimental conditions. Data analysis was conducted using GraphPad Prism 5 software. The error bars in the graphs represent the standard error of the mean.

## Data Availability

The authors confirm that the data supporting the findings of this study are available within the article.
